# HIV self-testing practices among Health Care Workers: feasibility and options for accelerating HIV testing services in Ethiopia

**DOI:** 10.11604/pamj.2013.15.50.2328

**Published:** 2013-06-09

**Authors:** Bekana Kebede, Tatek Abate, Desalew Mekonnen

**Affiliations:** 1 Department of Health Services Management, Institute of Public Health, University of Gondar, Ethiopia; 2 Department of Midwifery, College of Medicine and Health Sciences, University of Gondar, Ethiopia; 3 Department of Medicine, of Medicine and Health Sciences, University of Gondar, Ethiopia

**Keywords:** HIV, VCT, self-test, health care workers, Ethiopia

## Abstract

**Introduction:**

HIV is still an enormous global burden and it is also causing loss of huge health care workers (HCWs) on the already limited human resource capacity in health care services in Sub-Saharan Africa. Variety of methods of accelerating HIV testing is required to increase the rate of HIV testing and expand treatment services. Therefore, this study was aimed to find out the prevalence, feasibility and options of HIV self-testing practices in Ethiopia.

**Methods:**

A cross-sectional study design triangulated with qualitative method was conducted from February to May, 2012. The data was collected using a semi-structured pretested questionnaire and in-depth interview, at government and private health centers or clinics and hospitals. During the data collection all the available healthcare workers (HCWs) which encompass the internship students including: Medical, Health Officer, Nurses, Midwives and Laboratory students, and health professionals working in the selected health institutions were involved.

**Results:**

A total of 307 HCWs were included in the analysis and we found that 288(94.4%) of them were ever tested for HIV, of which majority 203 (70.5%) were tested by themselves though 244(80%) of the HCWs had motivation or interest to be tested by themselves. Generally, of the ever tested only 85(29.5%) were tested by the help of health care providers/counselors other than self. Regarding the place where the HCWs had the test, majority 136 (69.4%) tested by themselves at the health facility and the rest were tested at their home, office, market and church. The main reason stated for self-testing was the need for confidentiality for the test result, which was mentioned by 205(82%). Moreover, 35(14.0%) claims lack of time to access the ordinary counseling and testing services.

**Conclusion:**

This study depicts high rate of HIV self-testing practice among HCWs. This shows that HIV self-testing can be considered as one pillar to increase the HIV-testing services and a means for the HIV prevention and control policy, through increasing HIV testing uptake and awareness of HIV status. However, the implementation may require the role of different stakeholders and decision makers with further study to extend the options.

## Introduction

Globally in 2009, there were an estimated 2.6 million people became newly infected with HIV where more than 5 million people are now receiving HIV treatment. Ethiopia has one of the biggest shares of HIV epidemics in sub-Saharan Africa [1].

On the already limited human resource capacity in health care workforce HIV is also causing loss of health professionals. Africa, in particular, faces the loss of vast numbers of educated or trained health workers [2] and a recent study suggests that African health systems may lose 20% of their workers to HIV/AIDS over the coming years [3]. Study among South African health workers showed that in a sample of 721 HCWs an estimated 15.7% of HCWs employed in the public and private health facilities were living with HIV/AIDS [4] and one out of 7 nurses and nursing students in this public sector workforce was HIV-positive [5]. In Swaziland, 3 - 4% of nurses are lost to HIV infection annually [6] and in Kenya HIV incidence among health workers is twice the national average [7]. Data from Zimbabwe, Mozambique, Malawi, Kenya and Ethiopia indicate that 43% of deaths or medical retirement of health workers were suspected or known to be caused by HIV [8]. In South Africa, HIV prevalence among health workers was estimated to be 16 percent, at a time when it was estimated at 22 percent among the general population [9].

All HCWs has double burden in acquisition of HIV in that, they may face frequent work place blood or fluid contact and sharps injuries. WHO estimates that 2.5% of HIV infections among HCWs are a result of needle-stick injury [10]. Recent study conducted in Gondar, Ethiopia also have shown the prevalence of needle stick and sharp injuries among HCWs 12 months preceding the survey was 106(30.8%), of which 58(54.7%) was reported by females and highest being in nurses however, early post-exposure prophylaxis (PEP) uptake was reported as being low [11]. A study in Kenya found that only 4% of HCWs accessed PEP following needle stick injuries [12]. Fear of disclosure and reluctance to test may be major obstacles to HCWs access to HIV testing and counseling (HTC) and other related services [13, 14]. Being infected with HIV can be a source of personal and professional shame for a health worker, and may also invoke fear of losing one's job and damaging future career prospects [14–16]. Regardless of the outcome, it has been documented that having HIV test can be stigmatizing, and health workers are sometimes assumed to be HIV-positive if they are known to have been tested [16, 17].

A combination of strategically selected delivery modes by a variety of approaches is important to increase the HIV testing and access to treatment among HCWs and the community. However, according to WHO,HIV testing and counseling, regardless of the model of service delivery, must adhere to the five Cs — Consent, Confidentiality, Counseling, Correct test results and linkage to Care [18] though mandatory or coerced testing is never appropriate, whether that coercion comes from a health-care provider or from a partner or family member.

Reasons for self-testing are various, and exploring how to better addresses concerns around access, privacy, confidentiality. Potentially, self-testing for HIV offers individuals the opportunity to test for HIV at a time and place they prefer and offers complete privacy to those concerned about confidentiality [18, 21].

Some recent studies suggest that self-testing has the potential to be an innovative component to community-wide HIV prevention strategies [17, 19]. But, many policymakers have reservations about the introduction of self-testing and it is not currently widely available [20]. There is also increasing evidence that self-testing is practiced among HCWs; however, to date there is limited evidence on the feasibility and acceptability of HIV self-testing. High levels of informal's self-testing have been reported among health workers in sub-Saharan Africa [15]. This study would help as input information for policy recommendation of options for HIV-testing practices.

## Methods

A cross-sectional study design triangulated with qualitative method was conducted from February to May, 2012 among HCWs working at three government health centers (Gondar, Woreta and Addis Zemen Health Centers), three government hospitals namely Gondar University Hospital, Metema Hospital and Debark Hospital, and four private health facilities (three clinics & one General hospital) found in North and South Gondar Zones of Ethiopia.

Pretested semi-structured self-administered questionnaires were used to collect data from 307 HCWs. Besides, an in-depth interview was employed to collect the qualitative data at the health facilities by the investigators. Data was collected from all available and volunteer HCWs from aforementioned health facilities. The participants involved in the study were both employed HCWs and internship students practicing in the hospitals or health centers including; Medical Doctors, Health Officers, Nurses, Midwives, Laboratory Technicians and Pharmacists. The self administered semi-structured questioners were provided to the study participants independently in the form of exam while they were sitting for morning session with in their respective health institutions for the purpose of confidentiality and to obtain their full response. The study questionnaire addressed the socio-demographic factors, education and work experiences, history of risky behavior for HIV, occupational related injury, practice or experience of HIV testing, respondent's reason for self testing and challenges, and the way forward.

Finally, the data collected were cleaned for their internal consistency and entered into SPSS version 20.0 statistical software for analysis. Descriptive statistics were computed and described by graph and tables. The qualitative data were coded and analyzed thematically.

Ethical clearance was obtained from the University of Gondar, College of Medicine and Health Sciences Ethical Review Board. Official permission was obtained from each Health Institution supervisors. In addition, following discussion of the objective of the study, the study participants gave their informed consent. All participation was voluntary and the data collected was kept confidential by protecting the anonymity of respondents’ information.

## Results

### Characteristics of the study participants

A total of 307 HCWs were included in the analysis, majority (32.2%) were nurses by profession followed by Medical Doctors (11.1%). Most of the respondents 199(64.8%) were working at the Hospitals. Over seventy nine percent of the respondents were found at the age below 30 years and three fourth were having less than five years work experience. Regarding to the risk factors predisposing to HIV infection, 111(63.7%) of participants were reported history of risky sexual behavior; whereas 217(70.9%) notified history of occupational related risks like contacts with body fluids and sharp injuries (Table 1).

**Table 1:** HIV self-testing practices with the HCWs baseline characteristics of the respondents, Northwest Ethiopia

	Characteristics	Frequency (%)	Ever tested for HIV	Undergo HIV self-testing	
				No	Yes
			Number	Number (%)	Number (%)
Type of facility	Hospital	199 (64.8)	184	56(30.4)	128(69.6)
	Health center	79(25.7)	77	25(32.5)	52(67.5)
	Private clinics/hospital	29(9.4)	27	4(14.8)	23(85.2)
Age	Below 30 years	243(79.2)	228	55(24.1)	173(75.9)
	Above 30 years	64(20.8)	60	30(50.0)	30(50.0)
sex	Male	163(53.1)	151	49(32.5)	102(67.5)
	Female	144(46.9)	137	36(26.3)	101(73.7)
Marital status	Single	201(65.5)	186	61(32.8)	122(67.2)
	Married	96(31.3)	92	22(23.9)	70(76.1)
	Divorced	10(3.3)	10	2(20.0)	8(80.0)
Religion	Orthodox	239(78.1)	225	64(28.4)	161(71.6)
	Muslim	29(9.5)	26	4(15.4)	22(84.6)
	Protestant	30(9.8)	29	13(44.8)	16(55.2)
	Others(catholic, Adventist)	8(2.6)	8	4(50.0)	4(50.0)
Years of services	5 years or below	234(76.2)	219	62(28.3)	157(71.7)
	6 to 10 years	40(13.0)	38	12(31.6)	26(68.4)
	11 to 20 years	10(3.3)	10	4(40.0)	6(60.0)
	Above 21 years	23(7.5)	21	7(33.3)	14(66.7)
Profession	Medical Doctor/MD	34(11.1)	34	23(67.6)	11(32.4)
	Health Officer	13(4.2)	13	6(46.2)	7(53.8)
	Nurse	99(32.2)	92	16(17.4)	78(82.6)
	Laboratory technician	65(21.2)	65	14(21.5)	51(78.5)
	Midwife	34(11.1)	34	2(5.9)	32(94.1)
	Technical assistant	4(1.3)	4	4(100.0)	0(0.0)
	Internship/Graduating student	47(15.3)	37	16(43.2)	21(56.8)
	Pharmacy	8(2.9)	7	5(71.4)	2(28.6)
Educational status	Diploma	120(39.1)	115	19(16.5)	96(83.5)
	First Degree	171(55.7)	159	57(35.8)	102(64.2)
	Second degree/specialty	14(4.6)	12	7(58.3)	5(41.7)
History of risky sexual behavior	Yes	111(63.7)	179	28(25.7)	81(74.3)
	No	195(36.3)	109	57(31.8)	122(68.2)
History of occupational related injury	Yes	217(29.1)	80	59(28.4)	149(71.6)
	No	90(70.9)	208	26(32.5)	54(67.5)

### HIV testing practice among HCWs

The study revealed that 288(94.4%) of respondents were ever tested for HIV of which majority 203 (70.5%) were tested by themselves. Indeed, 244(80%) of the HCWs had motivation or interest to be tested by themselves. A total of 62.8% were tested themselves at least once or twice and, 28% were tested three to five times.

Of the ever tested, only 85(29.5%) were tested by the help of health care providers/counselors. The HCWs were reported that 16.1%, 22.4%, 20.3%, 16.4% and 24.8% had ever tested once, two times, three times, four to five times and more than five times, respectively (Table 2). Self-testing practices were more common in private health facilities followed by government hospitals, where (85.2%) and (69.6%) of them tested for HIV by themselves, respectively. Proportionally, female HCWs practice self-testing relatively similar to male (73.7%Vs 67.5%).Undergoing HIV self-testing was higher among midwifery nurse professionals than medical doctors (94.1% Vs 32.4%), and more among diploma holders (83.5%) than other HCWs with different educational level.

**Table 2:** HIV-testing practice of health care workers in Northwest Ethiopia

Factors	Number	Percent (%)
Have you ever tested for HIV		
Yes	288	94.4
No	17	5.6
How many times you have been previously tested for HIV?		
Once	46	16.1
One to two times	64	22.4
Three times	58	20.3
Three to five times	47	16.4
More than five times	71	24.8
What makes you to be tested for HIV?		
Simply to check my self	112	39.0
After initiated by counselor	28	9.8
Because of exposure to risky behavior and/or occupational injury	147	51.2
Have you motivation/interest for HIV self-testing?		
Yes	244	80.0
No	61	20.0
Who tested you for HIV previously?		
Myself only	80	27.8
Other person/health professional counselor	85	29.5
Both myself and also by counselor	123	42.7
How many times you have ever attempted self-test?		
One or two times	128	62.8
Three to five times	58	28.0
Six or more times	19	9.2
What was your feeling after you tested yourself?		
Nervous and sweating	60	30.9
I felt nothing	104	53.6
I immediately gone to health facility to check myself by others	30	15.5
If you were not practiced self-testing previously, why not?		
I don't have the skill	11	9.9
Testing materials were inaccessible	28	25.3
It is unethical and not allowed	41	36.9
I fear to test myself	31	27.9
Did you disclose your result after self-test?		
Yes	153	73.6
No	55	26.4
Did you refer yourself to health facility after self-test?		
Yes	41	20.0
No	160	80.0
Do you think counseling is necessary /required of the health care workers for HIV-testing?		
Yes	227	77.2
No	67	22.8
It what way counseling should be provided?		
Ordinary counseling available	113	39.0
Free-toll phone	35	12.2
Separated counseling and testing space should be available	84	28.8
Counseling should be given by external body	58	20.0
Did your partner also self-tested?		
Yes	143	49.8
No	144	50.2
Where did your partner tested?		
Home	57	39.9
Health facility	75	52.4
Office	9	6.3
Market	2	1.4
Do you recommend and prefer HIV self -test in future?		
Yes	195	66.8
No	97	33.2
What problems may occur if self-testing practiced?		
Inaccurate result	98	33.3
Unsafe disposal of sharps	30	13.6
Limited onward referral	15	5.1
Resource misuse	58	19.8
They may harm themselves after test result	83	28.2

Regarding to the place where the HCWs had the test, majority 136 (69.4%) were tested by themselves at the health facility and the rest 32(16.3%), 21(11.2%), 4(2.0%) and 2(1.0%) were tested at their home, office, market and church, respectively. The main reason stated for self-testing was the need for confidentiality for the test result which was mentioned by 205(82%) of the respondents. Moreover, 35(14.0%) claims lack of time to access the ordinary counseling and testing services.

In addition, while 22.2% of respondents claimed that counseling is not necessary for HCWs, 28.8% and 20% of the respondents recommended separated counseling and testing space should be available, and counseling should be provided by external body for the HCWs, respectively. Out of those health care workers who ever tested, three quarters (71.3%) of them recommended HIV self-testing for future, though some of them raised challenges including, 33.3% said inaccurate results may occur, 19.8% said resource misuse may occur and 28.2% raised the clients may harm themselves.

## Discussion

Uptake of HIV testing is almost universal and similar among HCWs in different health facilities. However, it is noteworthy that, 64.8% of them tested for HIV at least to the recent one year and the rest tested during last one to three months, in spite of the fact that these population segment are likely to be at a constant risk of work related HIV exposure and require frequent check-up for HIV.

Self-testing practices were more common in private health facilities followed by government hospitals. This implies that there may be restriction in access to test kits in the government health facilities and/or barriers to access the ordinary voluntary Counseling and Testing (VCT) services for employees in the private facilities.

In spite of the fact that 80% had motivation or the interest to test themselves, majority 70.5% were tested by themselves though not legally allowed, indicating presence of unmet need for self-test.

*“Well, as to me it is better especially for the health care workers because most of them of course have undergone the HIV testing for the issues of confidentiality and time barrier, many of them feel better by testing themselves. In addition those with higher educational level would not be tested by the one with lower education level. Medical doctors or health officers may undermine the counseling and testing of the nurses or other counselors. I have worked here in hospital VCT center for long time but never seen a single MD or HO who came to my VCT for counseling and testing. Thus allowing self-testing alternatively is good for HCWs“.* By Counselor Nurse

Three fourth of those HCWs who had faced risky occupational related injury were checked for HIV by themselves of which half of them were then tested again to be checked by the other health care provider or counselor. In some cases (42.7%), they were tested both by themselves and then also by the other HCWs. However, this was revealed by the in-depth interview that in majority they had first known their HIV status by them self before they repeated testing by the other health care professional counselor. It can be speculated that this may be due to fear of stigma.

*“Also, health care workers from very remote health centers and hospital come to our VCT center frequently because of their fear to be tested at their own health facility. In addition, one upon a time, one health professional come in troubled to be tested at our VCT center and later after i undergo testing he told me as he has tested himself but in doubt of the result”.* By Nurse

*“Most will not be tested by the counselor at the same working health institution, unless he/she first have known or checked him/herself and know his/her HIV status or he/she will be tested at different far health facility; this is common practice for our workers”.* By Health Officer.

Indeed, repeat testing was still useful in these individuals as it can be considered as they were disclosed their HIV status to their partners or other HCWs or referred themselves for testing as initiated by testing themselves. This concept was also revealed in study home-based VCT in Kenya [21].

**Figure 1:**
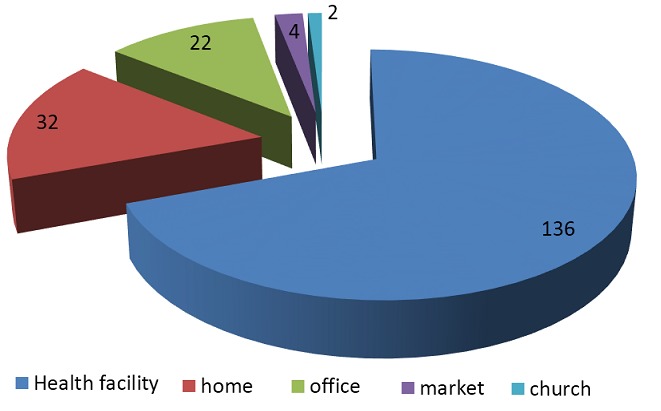
Place where HCWs practiced HIV self-testing in Northwest Ethiopia

Significant number (69%) of them attempted self-testing at their own health facility, while others did at their home (Figure 1). This finding supports an earlier study from Kenya which has indicated home-based HIV testing is feasible with high uptake potential to substantially expand access to HIV testing services [22].

The majority of those who self-tested disclosed or discussed the test results with someone (colleague, partner, and family). However, most of them didn't refer themselves to health facility VCT center for additional confirmatory testing.

This study also reported that about half of the HCWs partners were also practiced HIV self-testing, where one fourth of them did at their home. This also indicates a hope that the family based or home-based HIV-testing can be performed provided that there is adequate development of skill and counseling services. However, many of them raised its disadvantage.

*“Of course it is good if all professionals especially, know the HIV status of their family, however I doubt it may cause family distortion, and one may even harm him/her self if the result is not good due to lack of psychological support and good counseling. Thus I feel it will be better if we first think of how we can deliver appropriate counseling and support”.* By medical doctor

**Figure 2:**
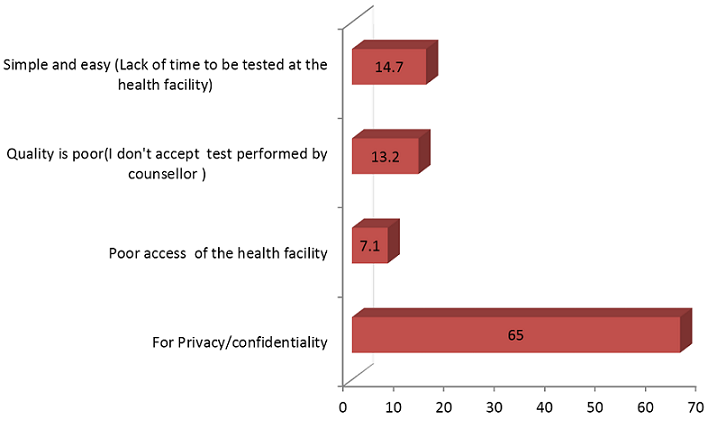
Reasons for self-testing practices among HCWs in Northwest Ethiopia

Self-testing can be radically an alternative to voluntary counseling and testing (VCT) and is attractive for many reasons. Self-testing may promises more confidentiality, more privacy, and ultimately, the chance for a greater number of people knows their HIV status. This study finding showed that, the reason for preferring self testing presented by most of HCWs 128(65%) was for privacy of test result (Figure 2) as similar to other studies[23].This was also supported by the in-depth-interview report:

*“Unless I first have known my HIV status how I could be tested here by my colleagues? In case if my test result turn positive I will be discriminated/stigmatized even I may be resigned from the health center. It is due to this most health workers find the need to travel to distant places for VCT as they feel that VCT at their own station would be not be confidential enough for them. Even not only me and my colleagues HCWs, our patients will go far long distant, other health facilities for their HIV care/ART to avoid stigma”.* By nurse

In relation to the mode of counseling for the HCWs, some respondents claimed that counseling is not necessary for HCWs, and others recommended separated counseling and testing space should be available, and counseling should be provided by external body for the HCWs. This is not in line with the WHO modes of service delivery; where counseling and consent should be given as models of HIV-testing regardless of the client's occupation or educational level [18]. However mandatory and coercive testing will not be appropriate still.

Out of those health care workers who ever tested, three quarters of them recommended HIV self-testing for future, though some of them raised challenges including; inaccurate results may occur, resource misuse and the clients may harm themselves. The result also indicated a significant number of the HCWs tested themselves frequently or abused of testing themselves. This could get attention of decision makers, health professional or policy makers to further the awareness and education of health professions on HIV testing procedures.

*“That is good way of course; many of my health center and outside colleagues asked me frequently to give them the HIV testing kits. In addition, once up on a time even we were facing frequently test kits stolen”.* by Laboratory Technician and Medical Doctor

## Conclusion

This survey revealed that there is high rate of HIV self-check practices among health care workers and most of them did at the respective health care institution. It shows that HIV self- testing can be considered as one options to increase the HIV-testing as one of the HIV prevention control policy and to increase the HIV-testing uptake of health care workers. The health care workers were tested themselves at their health facility, home, and offices. The main reason behind the need of self-testing was the need for confidentiality and lack of time for the ordinary VCT center which indicates that self-testing can reduce time burden to the health care workers. In addition the ordinary VCT service is not accessible to all population segments. Significant number of them were also tested their partners at their home, health facility or office which will also show the possibility of home-based, family based HIV-testing. Majority of the health care workers who ever self-tested were recommending self-checking of HIV in future as one pillar to expand access to the services. However, the implementation may require the role of different stakeholders and decision makers to extend the options to overcome the main shortcomings of self-testing raised by the participants. Implementation and scale-up of this program should be considered based on the lessons learned from this study and there is a need of addressing some questions or problems related with further studies.

## References

[1] UNAIDS report on the Global AIDS report 2010. http://www.unaids.org/documents/20101123_globalreport_em.pdf.

[2] World Health Organization The impact of HIV/AIDS on the health work force in developing countries; the world health report 2006.

[3] Joint Learning Initiative (2004). Human Resources for Health: Overcoming the Crisis.

[4] Shisana O, Hall EJ, Maluleke R, Chauveau J, Schwabe C (2004). HIV/AIDS prevalence among South African health workers. S Afr Med J..

[5] Connelly D, Veriava Y, Roberts S, Tsotetsi J, Jordan A (2007). Prevalence of HIV infection and median CD4 counts among health care workers in South Africa. S Afr Med J..

[6] Kober K, Van Damme W (2006). Public sector nurses in Swaziland: can the downturn be reversed?. Hum Resour Health..

[7] Tafwik S, Picazo O (2003). The impact of HIV/AIDS on health systems and health workforce in sub-Saharan Africa.

[8] Taegtmeyer M, Suckling RM, Nguku PM (2008). Working with risk: occupational safety issues among healthcare workers in Kenya. AIDS Care..

[9] Kalibala Sam, Waimar Tun, William Muraah, Peter Cherutich, Erick Oweya, Patricia Oluoch (2011). Knowing myself first?: Feasibility of self-testing among health workers in Kenya?.

[10] Kebede G, Molla M, Sharma HR (2012). Needle stick and sharps injuries among health care workers in Gondar city. Ethiopia Journal of Safety Science..

[11] Young TN, Arens FJ, Kennedy GE, Laurie JW, Rutherford GW (2007). Antiretroviral post-exposure prophylaxis (PEP) for occupational HIV exposure. Cochrane Database Syst Rev..

[12] Campbell S, Klein R (2006). Home testing to detect human immunodeficiency virus: boon or bane?. J Clin Microbiol..

[13] van Oosterhout JJ, Nyirenda M, Beadsworth MB (2007). Challenges in HIV post-exposure prophylaxis for occupational injuries in a large teaching hospital in Malawi. Trop Doct..

[14] World Health Organization (2006). Treat, Train, Retain: the AIDS and health workforce plan.

[15] Namakhoma I, Bongololo G, Bello G, Nyirenda L (2010). Negotiating multiple barriers: health workers’ access to counselling, testing and treatment in Malawi. AIDS Care..

[16] Corbett EL (2007). Health worker access to HIV/TB prevention, treatment and care services in Africa: situational analysis and mapping of routine and current best practices.

[17] Spielberg F, Levine RO, Weaver M (2004). Self-testing for HIV: a new option for HIV prevention?. Lancet Infect Dis..

[18] World Health Organization Service delivery approaches to HIV testing and counselling (HTC): a strategic HTC programme framework 2012.

[19] Choko AT, Desmond N, Webb EL, Chavula K (2011). The uptake and accuracy of oral kits for HIV self-testing in high HIV prevalence setting: a cross-sectional feasibility study in Blantyre, Malawi. PLoS Med..

[20] Lee VJ, Tan SC, Earnest A, Seong PS (2007). User acceptability and feasibility of self-testing with HIV rapid tests. J Acquir Immune Defic Syndr..

[21] World Health Organization HIV self-testing among health workers; a review of the literature and discussion of current practices, issues and options for increasing access to HIV testing in sub-saharan Africa WHO 2011.

[22] Negin J, Wariero J, Mutuo P, Jan S, Pronyk P (2009). Feasibility, acceptability and cost of home-based HIV testing in rural Kenya. Trop Med Int Health..

[23] Kachroo S (2006). Promoting self-testing for HIV in developing countries: potential benefits and pitfalls. Bull World Health Organ..

